# Dietary advanced glycation end products are associated with an increased risk of breast cancer in Iranian adults

**DOI:** 10.1186/s12885-023-11462-5

**Published:** 2023-10-03

**Authors:** Mitra Kazemi Jahromi, Asal Neshatbini Tehrani, Hossein Farhadnejad, Hadi Emamat, Hamid Ahmadirad, Farshad Teymoori, Zeinab Heidari, Niloufar Saber, Bahram Rashidkhani, Parvin Mirmiran

**Affiliations:** 1 Endocrinology and Metabolism Research Center, Hormozgan University of Medical Sciences, Bandar Abbas, Iran; 2 Student Research Committee, Ahvaz Jundishapur University of Medical Sciences, Ahvaz, Iran; 3 Department of Nutrition, School of Allied Medical Sciences, Ahvaz Jundishapur University of Medical Sciences, Ahvaz, Iran; 4 Nutrition and Endocrine Research Center, Research Institute for Endocrine Sciences, Shahid Beheshti University of Medical Sciences, Tehran, Iran; 5 The Persian Gulf Tropical Medicine Research Center, The Persian Gulf Biomedical Sciences Research Institute, Bushehr University of Medical Sciences, Bushehr, Iran; 6 Department of Nutrition, School of Public Health, Iran University of Medical Sciences, Tehran, Iran; 7 Department of Community Nutrition, Faculty of Nutrition Sciences and Food Technology, National Nutrition and Food Technology Research Institute, Shahid Beheshti University of Medical Sciences, Tehran, Iran

**Keywords:** Dietary advanced glycation end products, Dietary pattern, Cancer, Breast cancer, Women

## Abstract

**Background:**

Dietary advanced glycation end products (AGEs) can play an important role in increasing inflammatory factors and oxidative stress as risk factors for cancers. In the present study, we aimed to assess the relationship between dietary AGEs and the risk of breast cancer (BC) in Iranian adult women.

**Methods:**

This hospital-based case-control study includes 401 participants aged ≥ 30 years old. The cases group consisted of 134 women diagnosed with histologically confirmed BC. The control group included 267 women enrolled randomly from patients admitted to the same hospitals. Dietary intake information was determined using a validated food frequency questionnaire, and dietary AGEs intake was computed for all participants. Logistic regression models, adjusted for potential confounders, were used to determine the odds ratios (OR) and 95% confidence interval (CI) of BC across tertiles of dietary AGEs.

**Results:**

The mean ± SD age and body mass index of the study population were 47.92 ± 10.33 years and 29.43 ± 5.51 kg/m^2^, respectively. The median (interquartile) of dietary AGEs in all individuals was 9251(7450, 11,818) kU/day. After adjusting for age, first pregnancy age, and energy intake, participants in the highest tertile of dietary AGEs intakes had higher odds of BC compared to those in the lowest tertile of dietary AGEs (OR:2.29;95%CI:1.19–4.39, P_trend_:0.012). Additionally, in the multivariable model, after adjusting for age, age at first pregnancy, energy, menopausal status, family history of cancer, anti-inflammatory drug use, Vitamin D supplementation, physical activity, body mass index, number of childbirths, and history of abortion, breastfeeding, and oral contraceptive pills use, the odds of BC were increased across tertiles of dietary AGEs intake (OR: 2.33; 95%CI: 1.18–4.60, P_trend_: 0.017).

**Conclusion:**

The present findings suggest that a diet with high AGEs is associated with a higher likelihood of BC in adult women.

## Introduction

Breast cancer (BC) is the leading cause of global cancer incidence with 2.3 million new cases reported in 2020 accounting for one in six cancer deaths among women worldwide [1]. In Iran, BC is the most common cancer and the fifth cause of death related to cancers among women with a prevalence of 23.6% [2, 3]. The most important factors related to BC are age, genetic susceptibility, reproductive parameters, estrogen, elevated body mass index (BMI), and lifestyle [4, 5]. While genetic predisposition is important, up to 34% of BC cases can be prevented by modifying lifestyle factors such as physical activity, diet, alcohol consumption, and body weight [6].

Nutrition is one of the important and modifiable aspects of lifestyle that plays a role in the etiology of BC [7, 8]. A recent umbrella review found that higher consumption of red or processed meats, foods with a high glycemic index, and eggs were associated with a higher risk of BC, while the consumption of plant-based foods and nutrients such as calcium, vitamin D, folate, lignans, and carotenoids were inversely associated with BC risk [9]. Advanced glycation end products (AGEs) are other dietary components that their possible role in the pathogenesis of chronic diseases such as BC has recently warranted attention. These harmful products are mostly produced as a result of the thermal processing of animal-derived foods during food processing and cooking at high temperatures, such as deep frying, grilling, or broiling [10, 11]. AGEs are formed from the non-enzymatic reaction between reducing sugars and free amino groups of proteins, lipids, or nucleic acids and possess pro-oxidant properties in foods [12]. Carboxymethyl lysine (CML), which is mainly used as a dietary AGEs indicator, is one of the most important types of AGEs [13].

High intake of dietary AGEs has a positive effect on the serum level of these products in the body, aggravating inflammatory conditions and oxidative stress, and contributing to metabolic disorders [14–18]. In-vitro and in-vivo evidence and experimental studies show that AGEs can promote carcinogenesis and primary cancer cells through the formation of reactive oxygen species (ROS) and increased inflammation [19, 20]. Also, limited epidemiological studies have investigated the relationship between dietary AGEs and the risk of BCs [21, 22]; a large cohort study reported a 30% higher risk of developing BC in women consuming food with high content of dietary AGEs [21]. Also, another study on postmenopausal women found a 9% higher risk of BC in women who were in the highest quintile of AGEs intake [22].

It is important to note that the above-mentioned studies were conducted in Western countries, which have different eating habits and dietary patterns as well as different incidence rates of BC than developing countries in the Middle East and North Africa (MENA) region, such as Iran. In comparison to the USA and European countries, the incidence of BC in MENA region countries is lower, however, is steadily increasing. Also, BC patients in low-income countries still bear a greater burden of BC mortality [5, 23]. In the past decades, in European and North American countries, the common food patterns in individuals were rich in simple sugars, saturated fat, trans fatty acids, and sodium, which made them susceptible to chronic diseases, such as cancers; however, in recent years, the MENA region undergone a shift from the traditional healthy diet towards a more westernized diet rich in energy-dense foods, sweetened beverages, saturated fat, processed foods, and food items containing high levels of AGEs, which can affect the incidence of nutrition-related chronic diseases, such as cancers [24, 25]. Therefore, this study aimed to investigate the association between dietary AGEs intake and odds of BC in a sample of Iranian adult women, making it the first study in the MENA region to explore this relationship.

## Materials and methods

### Study population

This hospital-based case-control study was conducted at Shohada and Imam Hossain hospitals, which are referral hospitals in Tehran, Iran. The study was performed from September 2015 to February 2016. The case group consisted of newly diagnosed BC patients aged ≥ 30 years, who were selected from patients diagnosed with BC less than 6 months ago. Eligible participants in the case group included incident cases diagnosed with histologically confirmed BC in the past 6 months, who did not undergo any cancer treatments at the time of the interview, and who had proper general condition. Various exclusion criteria, including following special dietary habits such as vegetarianism, history of hormone replacement therapy (HRT), and being pregnant or lactating were considered for the selection of the final study population for the case group. For the control group, we also considered the various exclusion criteria, including history of HRT and benign breast disease, history of physician-diagnosed cancer in any site, pregnancy or lactating status, and following special dietary habits due to a particular disease or weight loss. Two controls were selected for each case in this study, and we randomly enrolled women admitted to the same hospitals, at the same time and same age (± 5year). The control group had been hospitalized mainly due to conditions such as traumas and orthopedic disorders, disk disorders, acute surgical conditions, and eye, ear, nose, or skin disorders.

The participation rate for all invited patients was 92%, with a rate of 95% for the case group and 89% for the control group. Of the 408 eligible subjects considered for this study, seven subjects were excluded as their energy intake was away from ± 3 standard deviations (SD) of the mean energy intake of the population. Among these excluded subjects, two were from the case group and five were from the control group. Finally, 401 women (134 cases and 267 controls) were included in the final analysis.

### Dietary assessment

Dietary intake data for cases and controls were collected by a trained dietician using a valid and reliable semi-quantitative 168-item food frequency questionnaire (FFQ) during a face-to-face interview [26]. To assess dietary intakes, a trained dietician asked participants that provide their daily, weekly, monthly, or yearly consumption frequency for each food item according to the standard portion size; then, these frequencies were transformed into grams per day using the household measure [27]. The participants’ energy, macronutrients, and micronutrient intake were computed using the USDA food composition table (FCT). For traditional food items that did not exist in the USDA database, we used Iranian FCT [28].

### Calculation of dietary AGEs intake

The dietary AGEs intake of participants (kU/day) was calculated using AGEs content in 108 food items from the 168-FFQ based on the studies conducted by Goldberg et al. [13] and Uribarry et al. [10]. To measure the total AGEs intake, we calculated the AGEs content (kU/100 g) of each food item in our FFQ based on these studies. For some traditional foods, we considered similar items found in these above-mentioned studies. Finally, we summed the AGEs value of all food items for each person as their total daily dietary AGEs intake.

### Assessment of non-dietary exposures

Participants’ weight was measured with minimal clothes and without shoes with a digital scale (Seca, Germany) with an accuracy of 100 g. We also measured the height of the participants in a standing position without shoes using a stadiometer to the nearest 0.5 cm. BMI was calculated by dividing weight (kg) by height squared (m^2^).

An expert nutritionist used a standard questionnaire to collect data on various variables, including age, smoking, education level, marital status, age at menarche, age at first pregnancy, abortion history, number of live births, menopausal status, breastfeeding history, history of hormone replacement therapy, history of oral contraceptive pills (OCP) use, benign breast diseases history, cancer family history, bra-wearing habits, family history of BC, supplement intake, and anti-inflammatory medication use. Information on physical activity (PA) levels was obtained by a valid and reliable questionnaire [29] and reported as Metabolic Equivalents per week (METs/week) [30, 31].

We determined participants’ socioeconomic status (SES) score [32] based on five dichotomous variables scored as 0 or 1: education (academic = 1, non-academic education = 0), family size (≤ 4 people = 1, >4 people = 0), income (high = 1, moderate and low = 0), house acquisition (house ownership = 1, without personal housing = 0), and foreign travel (yes = 1, no = 0). We then calculated the total SES score by summing the assigned scores (minimum SES score of 0 to maximum score of 5). An SES score of 3, 4, and 5 was categorized as high, 2 as moderate, and 1 or 0 as low.

### Statistical analysis

Statistical analysis was performed using Statistical Package Software for Social Science, version 21 (SPSS Inc., Chicago, IL, USA). The Student’s t-test and analysis of variance (ANOVA) were used to compare mean values for normally distributed variables, while the Mann–Whitney U test and Kruskal–Wallis test were used to compare variables without a normal distribution. The Chi-square test was used to compare the differences between cases and controls for categorical variables. Participants were categorized into tertiles based on cut points of dietary AGEs intake. Logistic regression was used to calculate the odds ratio (OR) and 95% confidence interval (95% CI) of BC according to the tertiles of the dietary AGEs intake. The multivariable logistic regression model was adjusted for age, age at first pregnancy, energy, menopausal status, family history of cancer, anti-inflammatory drug use, Vitamin D supplementation, physical activity, BMI, number of childbirths, and history of abortion, breastfeeding, OCP use. Since the etiology of BC can vary greatly according to menopausal status, we also conducted a subgroup analysis based on menopausal status and assessed the association between AGEs (per increment of each SD) and odds of BC in the pre-and post-menopausal women. All P-values are two-sided, and P-values < 0.05 were considered significant.

## Results

The mean age and BMI of all participants were 47.92 ± 10.33 years and 29.43 ± 5.51 kg/m^2^, respectively. Among the case and control groups, the mean age was 49.50 ± 10.67 and 47.13 ± 10.08 years, respectively. The median (interquartile) intake of dietary AGEs was 9451 (7803–11,855) kU/day for the case group and 9134 (7178–11,786) kU/day for the control group. The median intake of dietary AGEs across tertiles was 6594, 9125, and 13,628 kU/day, respectively.

Table 1 shows the study population characteristics based on the tertiles of dietary AGEs. Our findings indicated that the mean age of participants in the third tertile of AGEs was lower than those in the first tertile. However, there was no significant difference in various variables, including first pregnancy age, BMI, PA, smoking, marital status, menopausal status, cancer family history, anti-inflammatory drugs, breastfeeding history, education level, occupation status, family size, and SES across tertiles of dietary AGEs intake. Also, according to Table 1, the intakes of energy, fat, PUFA, MUFA, and SFA increased across tertiles of dietary AGEs intake, whereas the intakes of carbohydrate, protein, and fiber decreased across tertiles of dietary AGEs intake.

**Table 1:** Study population characteristics based on the tertiles cut points of dietary advanced glycation end products

Variables	T1 (n = 125)	T2 (n = 143)	T3 (n = 133)	P for trend
Demographic data				
Age (year)	48.8 ± 18.0	48.7 ± 19.4	46.1 ± 9.8	**0.022**
First pregnancy age (year)	18.1 ± 7.3	19.4 ± 7.7	18.4 ± 8.5	0.957
Body mass index (Kg/m^2^)	29.8 ± 5.5	29.7 ± 5.6	28.7 ± 5.4	0.088
Physical activity ( MET/min/week)	32.4 ± 5.1	32.8 ± 5.4	33.1 ± 5.2	0.294
Smoking (yes, %)	4.0	4.2	1.5	0.394
Marital status (married, %)	73.0	77.5	81.1	0.231
Menopausal status (yes, %)	52.4	48.6	38.6	0.070
Cancer family history (yes, %)	21.0	25.4	25.0	0.643
Anti-inflammatory drug (yes, %)	14.5	14.1	13.6	0.984
Vitamin D supplement intake (yes, %)	25.0	14.1	25.0	**0.032**
Breastfeeding history (yes, %)	85.5	87.3	84.8	0.827
Education level (Bachelor and higher, %)	14.5	16.2	18.2	0.698
Occupation (employed, %)	21.8	14.8	22.7	0.180
Family size (> 4 members, %,)	51.6	54.9	60.6	0.338
Socio economic status (%)				0.533
Low (%)	40.3	40.1	31.8	
Middle (%)	41.1	40.1	43.2	
High (%)	18.5	17.6	23.5	
**Dietary intake**				
Energy intake(Kcal/d)	2237 ± 483	2572 ± 561	3249 ± 781	**< 0.001**
Carbohydrate (% of energy)	56.2 ± 5.2	53.0 ± 5.5	49.2 ± 6.2	**< 0.001**
protein(% of energy)	13.0 ± 1.8	12.9 ± 2.1	12.0 ± 2.2	**< 0.001**
fat(% of energy)	30.8 ± 5.0	34.0 ± 5.6	38.7 ± 6.9	**< 0.001**
Polyunsaturated fatty acids(% of energy)	6.5 ± 2.2	7.7 ± 2.4	9.2 ± 3.2	**< 0.001**
Monounsaturated fatty acids(% of energy)	10.5 ± 2.3	11.9 ± 2.4	13.6 ± 3.3	**< 0.001**
Saturated fatty acids(% of energy)	9.9 ± 2.0	10.6 ± 2.0	11.6 ± 2.4	**< 0.001**
Fiber (g/1000 Kcal)	14.7 ± 4.0	14.2 ± 5.1	13.2 ± 4.3	**0.007**

Data are expressed as mean ± SD and percent (%) for continuous and categorical variables, respectively

Table 2 reported the population characteristics, including demographic data, anthropometric measures, clinical features, and dietary intakes of BC patients (case group) and healthy participants (control group). The mean age and percentage of menopausal status, and cancer family history were higher in the case group compared to the control group, whereas the percentage of vitamin D supplement intake and anti-inflammatory drug use was lower in the case group compared to the control group. However, there was no significant difference between the case and control groups in first pregnancy age, mean BMI, PA level, smoking status, marital status, percentage of men, breastfeeding history, education level, occupation status, family size, and SES. Table 2 indicated that the energy intake was higher in the control group compared to the case group, but, no significant difference was observed in other dietary intakes between the two groups.

**Table 2:** Study population characteristics among the breast cancer patients and healthy participants

Variables	Control (n = 267)	Case (n = 134)	P-value
Demographic data			
Age (year)	47.1 ± 10.0	49.5 ± 10.7	**0.035**
First pregnancy age (year)	18.2 ± 7.4	19.6 ± 8.6	0.105
Body mass index (Kg/m^2^)	29.0 ± 5.4	30.1 ± 5.7	0.071
Physical activity ( MET/min/week)	32.7 ± 5.2	32.9 ± 5.4	0.701
Smoking (yes, %)	3.4	3.0	0.842
Marital status (married, %)	77.2	78.4	0.733
Menopausal status (yes, %)	42.7	53.7	**0.037**
Cancer family history (yes, %)	20.6	30.6	**0.028**
Anti-inflammatory drug (yes, %)	17.2	7.5	**0.007**
Vitamin D supplement intake (yes, %)	24.3	14.9	**0.029**
Breastfeeding history (yes, %)	86.1	85.8	0.930
Education level (Bachelor and higher, %)	14.6	19.4	0.178
Occupation (employed, %)	20.6	17.2	0.442
Family size (> 4 members, %,)	55.4	56.7	0.807
Socio economic status (%)			0.531
Low (%)	37.1	38.1	
Middle (%)	43.8	37.3	
High (%)	18.7	21.6	
**Dietary intake**			
Energy intake(Kcal/d)	2753 ± 798	2562 ± 612	**0.015**
Carbohydrate (% of energy)	53.0 ± 6.4	52.4 ± 6.1	0.437
protein(% of energy)	12.7 ± 2.1	12.4 ± 2.0	0.100
fat(% of energy)	34.3 ± 6.7	35.2 ± 6.6	0.213
Polyunsaturated fatty acids(% of energy)	7.7 ± 2.8	8.1 ± 2.8	0.119
Monounsaturated fatty acids(% of energy)	11.8 ± 3.0	12.4 ± 2.9	0.056
Saturated fatty acids(% of energy)	10.6 ± 2.4	10.9 ± 1.9	0.326
Fiber (g/1000 Kcal)	14.1 ± 4.7	13.9 ± 4.3	0.724
Dietary AGEs intake (kU/day)	10,150 ± 4265	10,017 ± 3275	0.752

Data are expressed as mean ± SD and percent (%) for continuous and categorical variables, respectively

The ORs and 95% CIs for BC across tertiles of AGEs are presented in Table 3. In the crude mode, there was no significant association between dietary AGEs intakes and risk of BC (OR: 1.22; 95%CI: 0.71–2.07, P for trend: 0.459). However, in model 2, after adjusting for age, first pregnancy age, and energy intake, the odds of BC increased noticeably across tertiles of the dietary AGEs intakes (OR: 2.29; 95%CI: 1.19–4.39, P for trend:0.012). Also, in the final model, an additional adjustment for menopausal status, family history of cancer, anti-inflammatory drug use, Vitamin D supplementation, physical activity, BMI, number of childbirths, and history of abortion, breastfeeding, and OCP use, the odds of BC in participants in the highest tertile of dietary AGEs intakes were higher than those in lowest tertile (OR: 2.33; 95%CI: 1.18–4.60, P for trend: 0.017).

**Table 3:** Odds ratios (ORs) and 95% confidence intervals (CIs) for breast cancer based on tertiles of dietary advanced glycation end products (AGEs)

	Tertiles of dietary advanced glycation end products			
	T1	T2	T3	P for trend
**Median score of dietary AGEs**	6594	9125	13,628	
**Case/Total**	36 / 125	53 / 143	44 / 133	
**Model 1** ^*^	1.00 (Ref)	1.47 (0.87–2.46)	1.22 (0.71–2.07)	0.459
**Model 2** ^**†**^	1.00 (Ref)	1.77 (1.03–3.06)	2.29 (1.19–4.39)	0.012
**Model 3** ^**‡**^	1.00 (Ref)	1.61 (0.92–2.81)	2.16 (1.11–4.18)	0.023
**Model 4** ^**¶**^	1.00 (Ref)	1.62 (0.92–2.85)	2.33 (1.18–4.60)	0.017

^*^Model 1: Crud model

^†^Model 2: Adjusted for age, first pregnancy age, and energy intake

^‡^ Model3: adjusted for model 2 and menopausal status, family history of cancer, anti-inflammatory drug use, Vitamin D supplementation, physical activity

^¶^ Model 4: adjusted for model 3 and BMI, number of childbirths, and history of abortion, breastfeeding, and OCP use

Table 4 reported the ORs and 95% CIs for BC based on tertiles of dietary AGEs from different food sources, including meats, dairy products, cereals, oil, vegetables, fruits, nuts, legumes, and sweets. In the fully adjusted regression model, after adjusting for age, first pregnancy age, energy intake, menopausal status, family history of cancer, anti-inflammatory drug use, Vitamin D supplementation, physical activity, BMI, number of childbirths, and history of abortion, breastfeeding, and OCP use, participants in the highest tertile of dietary AGEs from oil had a higher odds of BC than those in the lowest tertile (OR: 3.18; 95%CI: 1.69–5.98, P for trend < 0.001). Also, a direct relationship between dietary AGEs from meat (OR: 1.04), dairy products (OR: 1.49), sweets (OR: 1.17), and cereals (OR: 1.25), and the risk of BC was observed, although it was not statistically significant. The results regarding the association of dietary AGEs from fruits, vegetables, legumes, and nuts with the risk of BC were not statistically significant.

**Table 4:** Odds ratios (ORs) and 95% confidence intervals (CIs) for breast cancer based on tertiles of dietary advanced glycation end products (AGEs) from different food sources

	Tertiles of dietary advanced glycation end products from different food sources			
	T1	T2	T3	P-Value*
**AGEs of Meats**	1.00 (Ref)	0.64 (0.37–1.13)	1.04 (0.60–1.80)	0.900
**AGEs of dairy products**	1.00 (Ref)	1.35 (0.76–2.39)	1.49 (0.85–2.62)	0.160
**AGEs of cereals**	1.00 (Ref)	1.06 (0.61–1.85)	1.25 (0.69–2.24)	0.456
**AGEs of oils**	1.00 (Ref)	2.34 (1.31–4.19)	3.18 (1.69–5.98)	< 0.001
**AGEs of vegetables**	1.00 (Ref)	0.68 (0.39–1.20)	0.61 (33 − 1.10)	0.104
**AGEs of fruits**	1.00 (Ref)	0.67 (0.38–1.22)	0.80 (0.45–1.39)	0.200
**AGEs of nuts**	1.00 (Ref)	0.76 (0.44–1.40)	0.94 (0.51–1.69)	0.821
**AGEs of legumes**	1.00 (Ref)	0.52 (0.30–0.93)	0.67 (0.40–1.20)	0.185
**AGEs of sweets**	1.00 (Ref)	0.96 (0.55–1.66)	1.17 (0.66–2.04)	0.580

*Adjusted for age, first pregnancy age, energy intake, menopausal status, family history of cancer, anti-inflammatory drug use, Vitamin D supplementation, physical activity, BMI, number of childbirths, history of abortion, breastfeeding, and OCP use

Furthermore, in a subgroup analysis based on the menopausal status, we assessed the association of AGEs (per increment of each SD) and odds of BC in the pre-and post-menopause women. Table 5 showed that in post-menopause women, in the multi-variables model, there was a direct association between per increment of each SD dietary AGEs intake and increased odds of BC (OR: 1.70; 95%CI: 1.11–2.60, P for trend: 0.014). However, in pre-menopause women, no significant association was observed between dietary AGEs intake and odds of BC.

**Table 5:** Odds ratios (ORs) and 95% confidence intervals (CIs) for breast cancer per each SD increase in dietary advanced glycation end products (AGEs) based on menopausal status

	Pre-menopause		Post-menopause	
	OR (95% CI)	P-value	OR (95% CI)	P-value
**Z score of dietary AGEs**	41,950		3626	
**Case/Total**	62 / 215		72 / 186	
**Model 1** ^*^	0.84 (0.61–1.16)	0.300	1.17 (0.87–1.57)	0.285
**Model 2** ^**†**^	0.96 (0.64–1.43)	0.849	1.65 (1.11–2.46)	0.013
**Model 3** ^**‡**^	0.94 (0.62–1.44)	0.798	1.62 (1.08–2.43)	0.019
**Model 4** ^**¶**^	0.94 (0.61–1.46)	0. 803	1.70 (1.11–2.60)	0.014

^*^Model 1: Crud model

^†^Model 2: Adjusted for age, first pregnancy age, and energy intake

^‡^ Model3: adjusted for model 2 and family history of cancer, anti-inflammatory drug use, Vitamin D supplementation, physical activity

^¶^ Model 4: adjusted for model 3 and BMI, number of childbirths, and history of abortion, breastfeeding, and OCP use

## Discussion

The present study is the first study in Iranian women that showed a positive association between dietary AGEs intake and the risk of BC. The higher risk of BC across tertiles of dietary AGEs was strengthened after adjusting for various confounding factors.

Our finding is in line with the results from previous limited studies conducted on different populations, which assessed the possible association of dietary AGEs or its serum level with the risk of BC or its mortality [21, 22, 33, 34]. A prospective cohort study on 183,548 postmenopausal women showed a positive role of higher dietary AGEs intake in the progression of BC in the postmenopausal phase [22]. Similarly, Omofuma et al. reported that women with a higher intake of dietary AGEs may be more at risk of developing BC [21]. Also, according to the findings of a prospective women’s health initiative study, higher dietary AGEs intake may be related to increased premature death and mortality in postmenopausal women with BC [33]. Furthermore, Pan et al. have reported a positive correlation between serum AGEs levels and metastasis of BC [34]. Therefore, the results of the studies conducted in developed countries, as well as the findings of our study, support the suggestion that reducing the intake of dietary AGEs through dietary pattern modification as a main part of the lifestyle can be an important preventive action in women to reduce the risk of the occurrence of BC or prevention of its progress. This suggestion is also mentioned in the study by Walter et al., which reported that regarding the links between lifestyle, AGEs, and diseases, reducing the exposure to AGEs along with lifestyle modification were preventive and treatment options in patients with BC [35].

The adverse role of AGEs and their involvement as an important contributor to cancer initiation and advancement can be explained by some possible mechanisms. As mentioned earlier, AGEs production is the result of glycation and oxidation of proteins and lipids. Binding AGEs to their receptors (RAGE) leads to the activation of some signaling pathways, including nuclear factor (NF)-kB and phosphoinositide 3-kinase (PI3K)/Akt, which can contribute to the development of chronic diseases, such as cancers [36]. AGEs can also produce ROS, create free radicals, and be involved in auto-oxidation reactions [37]. At the cellular level, this can lead to an imbalance between ROS and antioxidant defense, which results in oxidative stress via the accumulation of free radicals. The free radicals cause structural modification in DNA, proteins, lipids, therefore, changing their biological features and, initiating tumor progression, and increasing the risk of cancers [38, 39]. Logsdon et al. showed that the expression of RAGE is highly elevated in breast tumors [40]. Additionally, the proliferation and invasion of BC cells are the results of enhanced expression of RAGE [41, 42].

Overall, various food items, consumed as part of an individual’s dietary pattern, can contribute to an increased pool of AGE within the body [10, 43, 44]. An increase in NƐ- CML as a marker of circulating AGEs has been observed in BC tissues [45], and serum levels of CML are elevated in women diagnosed with BC [34]. An unhealthy lifestyle, which includes a poor diet rich in sugar, animal protein, and fat, along with lower consumption of fruits, whole grains, and vegetables can lead to AGE accumulation and increase the risk of chronic diseases [10, 46–48]. Our study indicated that high intake of AGEs from oils can increase the chance of BC. On the other hand, although the results regarding the relationship between dietary AGEs intake from meat, cereals, and dairy products and the risk of BC were not statistically significant, the value of reported ORs (> 1.00) suggests that the cumulative effect of dietary intake of these food groups can make population susceptible to BC. Moreover, we found that a higher percentage of fat intake was associated with higher AGEs intake, consistent with the fact that foods with higher amounts of fat contain more AGEs [10]. Conversely, advancing in tertiles of dietary AGEs intake was associated with a lower percentage of carbohydrate consumption from total dietary energy intake. This may be due to the assumption that carbohydrates with higher water content and health-friendly agents such as vitamins and antioxidants have a lower amount of AGEs [10]. These findings are consistent with the Mirmiran et al. report [18], which showed the same result on the association between dietary AGEs intake and carbohydrates and fat intake.

Given the adverse effects of AGE intake on health, lifestyle modification, including increasing levels of exercise and adhering to a healthy diet, have been reported as effective strategies for reducing the level of circulating AGEs in patients with BC. To reduce the level of circulating AGEs in individuals and mitigate their detrimental effects, several recommendations can be prescribed by nutritionists and dieticians. These recommendations include increasing the consumption of food items with low AGEs content, such as whole grains, fruits, vegetables, legumes, seafood, and low-fat dairy, and also lowering the consumption of food items with higher AGEs content, such as sugar-added food items (i.e. candy, cookies, and beverages), highly processed foods (i.e. processed meats, packaged cheese, and snack-type foods) and high-fat foods (including butter, full-fat cheeses, and fried foods). In addition to dietary modifications, changing cooking methods, such as preferring those with low temperatures and high moisture (e.g., boiling, steaming, poaching, and stewing for a relatively long duration), can also help to reduce the level of AGEs in the body. Appropriate modification in lifestyle behaviors, such as increasing physical activity, reducing body weight, and avoiding smoking can also help to reduce the level of AGEs in the body and prevent possible damages caused by them [18, 49].

In the current study, according to baseline results, there was no significant difference in mean BMI according to the tertiles of dietary AGEs intake. Also, there was no significant difference in the mean BMI of the participants between the case and control groups. So, the baseline results for BMI level in Tables 1 and 2 showed that although the level of BMI in the participants is at a high level, it seems that its effect in predicting the risk of BC may be similar across different tertiles of AGEs intake in the current study. It is worth noting that, considering the evidence extracted from the literature review, high BMI is a key predicting factor in the risk of BC in women, and therefore, we adjusted the effect of BMI in the study analyses as an important confounder. Also, given that the role of BMI in predicting the risk of BC in pre-menopausal and post-menopausal women may be different [50–52], we performed a subgroup analysis of participants based on menopausal status. In the multi-variables adjusted model, we observed a direct association between per increment of each SD dietary AGEs intake and increased odds of BC in post-menopause women, however, in pre-menopause women, no significant association was observed between dietary AGEs intake and odds of BC. These findings are in line with the results of the previous investigation that reported an increased risk of BC with an increase in BMI in post-menopausal women [51–53]. It is suggested that in postmenopausal women, an increase in BMI is strongly related to an increase in fat mass, whereas it is unlikely that this individual is very muscular. Also, the high risk of BC among postmenopausal women with elevated BMI levels may mainly be associated with an increase in estrogens [53]; this hormonal response may stimulate more estrogen-sensitive breast tissues that may already have a propensity for hyperstimulation, subsequently play a possible role in the promotion of the formation and development of malignant cells [52, 54].

The nature of the case-control study design raises the possibility that individuals with breast cancer (BC) change their food intake and pattern after the diagnosis of their clinical problem. To avoid this bias, we included only newly diagnosed BC patients (new cases) in this study that did not have the opportunity to change their lifestyle, such as dietary pattern for BC management. It is also necessary to mention that we collected nutritional data in BC patients for the last year before the disease was diagnosed in them so that we can have a more appropriate and accurate estimation of their dietary pattern as an essential exposure for BC risk. One issue worth discussing is whether using an FFQ to collect dietary intake data for the last year of patients can show their dietary pattern for at least the last 5–10 years, which may have a significant role in predicting the risk of BC as an important part of an individual’s lifestyle; in response to this question, it should be explained that previous studies in nutritional epidemiology suggest that dietary intake does not change much in less than 5–10 years in adults [51], therefore, using an FFQ can provide an appropriate estimate of long term food intakes and dietary pattern of individuals [50], and allow us to determine the potential role of different aspects of dietary patterns in predicting chronic diseases risk (such as BC in the present study) after adjusting for the effect of confounding factors.

The present study has several strengths. It is the first study in the MENA region to investigate the relationship between dietary AGEs and the risk of BC in adult women. We used valid and reliable questionnaires for dietary intake and PA level assessments, which reduced measurement error. Also, we controlled for and adjusted the confounding effect of various variables in assessing the relationship between dietary AGEs and BC risk. We should consider several limitations as well. First, there is no database regarding the AGEs content of different Iranian foods, so the American database was used. Second, we did not collect data on different cooking methods that can affect the AGEs content of foods. Furthermore, the serum level of AGEs in the subjects was not available, which could have been helpful in estimating the possible effect of dietary AGEs intake on the serum level of AGEs in the study population. Because of religious considerations, alcohol drinking is unusual in the Iranian people, so we couldn’t collect data on alcohol consumption in participants, however, it should be noted that generally, alcohol consumption in women has a very low prevalence in our society. Due to the case-control nature of the study, reverse causality can somewhat occur. In the current study, similar to several other epidemiologic studies, the ELISA method has been used to measure AGEs in food samples. Although a vast body of literature supports the validity and reliability of this method, other methods, such as the ultra-performance liquid chromatography-tandem mass spectrometry (UPLC-MS/MS) approach, have been developed to determine dietary AGEs that may show greater accuracy in estimating their dietary content. The subjects selected for the case group may have made changes in nutritional and non-nutritional exposures (such as BMI and PA) under the influence of their disease; however, to minimize these limitations, the individuals selected for the case group were subjects who on newly (< 6 months) diagnosed with BC, therefore, it is expected these participants mostly have not noticeable changes in their nutritional or non-nutritional characteristics arbitrarily or based on the order of physicians. Moreover, the consideration of various inclusion and exclusion criteria has led to individuals in the case and control groups not being significantly different in most characteristics (as shown in Tables 1 and 2). The use of multivariable regression analysis, adjusted for the confounding effect of various variables, is another help to reduce these limitations in the present study. Finally, despite adjusting the effect of various confounders in the final analysis, the confounding effect of some variables that were not measured or known could not be controlled.

## Conclusions

Our results reported that a dietary pattern with high AGEs was associated with increased odds of BC in Iranian adult women. This is a very important result since it can help introduce a dietary pattern that focuses on food components with little or no AGEs, which can be easily adhered to by the public to prevent the occurrence of chronic diseases such as BC.

## Data Availability

The datasets analyzed in the current study are available from the corresponding author on reasonable request.

## Abbreviations

AGEs: Dietary advanced glycation end products
ANOVA: analysis of variance
BC: breast cancer
BMI: body mass index
CIs: confidence intervals
CML: carboxymethyl-lysine
FCT: Food Composition Table
FFQ: food frequency questionnaire
METs: Metabolic Equivalents
NF: nuclear factor
OCP: oral contraceptive pills
ORs: odds ratios
PA: physical activity
PI3K: phosphoinositide 3-kinase
RAGE: Receptor for AGE
SES: socioeconomic status
SPSS: Statistical Package Software for Social Science
USDA: United States Department of Agriculture
WHR: waist-hip ratio
